# Skin‐Inspired Capacitive Flexible Tactile Sensor with an Asymmetric Structure for Detecting Directional Shear Forces

**DOI:** 10.1002/advs.202305883

**Published:** 2023-12-07

**Authors:** Haibo Yu, Hongji Guo, Jingang Wang, Tianming Zhao, Wuhao Zou, Peilin Zhou, Zhuang Xu, Yuzhao Zhang, Jianchen Zheng, Ya Zhong, Xiaoduo Wang, Lianqing Liu

**Affiliations:** ^1^ State Key Laboratory of Robotics Shenyang Institute of Automation Chinese Academy of Sciences Shenyang 110016 China; ^2^ Institutes for Robotics and Intelligent Manufacturing Chinese Academy of Sciences Shenyang 110016 China; ^3^ University of Chinese Academy of Sciences Beijing 100049 China; ^4^ College of Mechanical and Electrical Engineering Henan Agricultural University Zhengzhou 450002 China

**Keywords:** asymmetric microhair array, capacitive, directional shear force, flexible tactile sensor, two‐photon polymerization

## Abstract

Flexible pressure sensors based on micro‐/nanostructures can be integrated into robots to achieve sensitive tactile perception. However, conventional symmetric structures, such as pyramids or hemispheres, can sense only the magnitude of a force and not its direction. In this study, a capacitive flexible tactile sensor inspired by skin structures and based on an asymmetric microhair structure array to perceive directional shear force is designed. Asymmetric microhair structures are obtained by two‐photon polymerization (TPP) and replication. Owing to the features of asymmetric microhair structures, different shear force directions result in different deformations. The designed device can determine the directions of both static and dynamic shear forces. Additionally, it exhibits large response scales ranging from 30 Pa to 300 kPa and maintains high stability even after 5000 cycles; the final relative capacitive change (Δ*C*/*C*_0_) is <2.5%. This flexible tactile sensor has the potential to improve the perception and manipulation ability of dexterous hands and enhance the intelligence of robots.

## Graphical abstract

Asymmetric microhair structures are obtained by two‐photon polymerization (TPP) and replication. The tactile sensor based on a simple asymmetric structure can sense shear forces without further signal processing. This flexible tactile sensor has the potential to improve the perception and manipulation ability of dexterous hands and enhance the intelligence of robots.

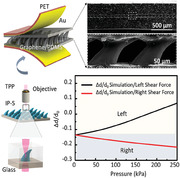

## 1 Introduction

Tactile sense helps humans perceive and understand their complex surroundings;^[^ ^1^, ^2^ ^]^ therefore, robots in unstructured environments, particularly those in the fields of smart prosthetics and human‒machine interfaces, must also have tactile sense.^[^ ^3^, ^4^, ^5^, ^6^, ^7^, ^8^, ^9^, ^10^ ^]^ For improving spatial interaction^[^ ^11^, ^12^ ^]^ and pressure sensitivity, multisensor arrays with symmetric structures,^[^ ^13^, ^14^, ^15^, ^16^, ^17^ ^]^ such as pyramids,^[^ ^18^ ^]^ hemispheres,^[^ ^19^ ^]^ or micropillars,^[^ ^20^ ^]^ can be integrated into the surface of an artificial limb as e‐skin to capture human–environment interactions and enhance the interactive ability and safety of robots.^[^ ^21^, ^22^ ^]^ However, tactile perceptions are induced not only by pressure but also by shear and frictional forces,^[^ ^23^, ^24^, ^25^ ^]^ whose direction and magnitude are both significant. Currently, the interest in detecting shear and frictional forces is increasing.^[^ ^26^, ^27^, ^28^, ^29^, ^30^, ^31^ ^]^ For instance, Zhou et al. used spine arrays as dielectric layers for flexible capacitive sensors that can detect both pressure and shear force.^[^ ^32^ ^]^ Zhang et al. designed a plane‐parallel capacitor as a tactile sensor, inspired by fingerprints and Ruffini endings, which can distinguish between static and sliding frictional forces.^[^ ^33^ ^]^ Bao et al. proposed an interlocked microstructure array with 25 pixels to provide sufficient information to measure tilted forces.^[^ ^34^ ^]^ Jung et al. developed a piezoresistive‐type tactile sensor based on a flexible core and four sidewall structures capable of determining both the direction and magnitude of forces.^[^ ^35^ ^]^ However, the development of tactile sensors with pixel array structures requires further signal processing, and the sidewall style requires more space. Therefore, a new type of tactile sensor based on a simple structure that does not require further signal processing must be developed.

Human skin is a complex tactile perception system with high sensitivity and integration.^[^ ^1^, ^36^ ^]^ Therefore, most biomimetic tactile sensors are inspired by skin structures such as mechanoreceptors and interlocked spinous layers.^[^ ^36^, ^37^, ^38^, ^39^, ^40^, ^41^, ^42^ ^]^ Human skin can perceive the direction of tiny stimulations owing to the presence of hair and the longitudinal lanceolate endings around the hair follicle.^[^ ^2^ ^]^ Similarly, hairs on arthropods act as stimulated‐information capturing devices: the biological tissue at the root of the hair acts as a transduction unit, whereas the nerve cells act as intelligent processing modules.^[^ ^38^ ^]^ Hair can distinguish directional stimulation to the transduction unit because of its asymmetric tilted structure. Hence, hair‐like structures provide a novel biomimetic approach for flexible tactile sensors for sensing shear and friction forces.

In this study, we proposed a capacitive tactile sensor with tilted microhair arrays (TMHAs), which have an asymmetric structure, with a hair‐like structure array as the dielectric layer. The TMHAs were fabricated using two‐photon polymerization (TPP) and replication processes; additionally, graphene was mixed with the dielectric layer to increase conductivity. This sensor can detect a pressure load of 30 Pa and respond to shear forces. Finally, to demonstrate the potential of the hair‐inspired capacitive tactile sensor, the devices were integrated into a dexterous hand as human–machine e‐skin to enhance tactile perception, safety, and responsiveness. The introduction of the shear force correction can guarantee the stability of operation. This study provides a new direction for tactile sensors and drives the development of human–machine interactions.

## 2 Results and Discussion

### 2.1 Design Principle and Fabrication Process of the Tilted Microhair Array

**Figure 1:**
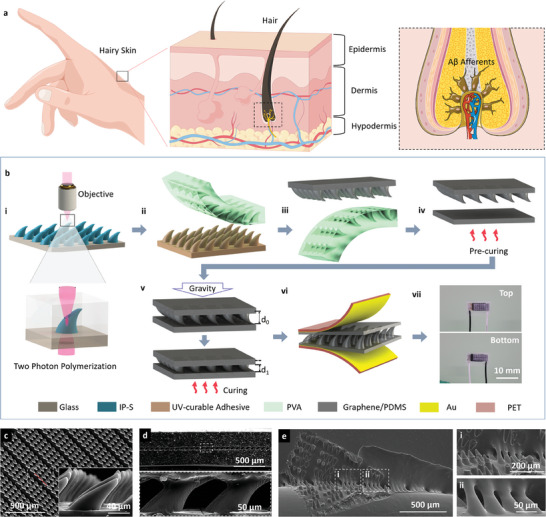
Biomimetic mechanism, fabrication process, and characterization of TMHA‐based tactile sensor. a) Perception mechanism of fine hairs. b) Fabrication process of TMHA‐based tactile sensor. c) SEM images of graphene/PDMS TMHA. d) Section SEM images of the TMHA‐based tactile sensor. e) SEM images of the devices after rough peeling. The enlarged images (i) and (ii) show the discrete rupture mode of TMHA that prevents continuous, catastrophic, and brittle failure.

We designed a TMHA structure inspired by skin configuration, to act as a dielectric layer for capacitive tactile sensors. Human skin is a comprehensive system containing the epidermis, dermis, and hypodermis, along with numerous mechanoreceptors, as shown in **Figure** **1**a. Hairs grow from hair follicles embedded in the skin, and tiny deformations are transmitted to Aβ afferents as tactile receptors to perceive pressure, shear, and friction forces. The hair responds to shear in different directions by producing corresponding deformations, which may be the key to distinguishing between the directional shears of the skin. The biased hair structure manifests bioinspired properties for fabricating TMHA‐based tactile sensors, as shown in Figure 1b. Herein, first, the TMHA structure was polymerized on a glass substrate using TPP systems with high accuracy (Figure 1b‐i). Subsequently, a robust TMHA‐based graphene/polydimethylsiloxane (PDMS) dielectric layer was obtained via four replication processes (Figure 1b‐ii,iii and Figures S1 and S2, Supporting Information). Finally, a precured graphene/PDMS layer was placed under the TMHA‐based graphene/PDMS layer at 100 °C for 20 min (Figure 1b‐iv,v). A polyethylene terephthalate (PET) film sputtered with gold was used as the electrode layer (Figure 1b‐vi), and the package sensor measured 5 × 6 mm^2^ (Figure 1b‐vii). Owing to gravity and the all‐graphene‐PDMS material system, the TMHA‐based graphene/PDMS and graphene/PDMS electric layers form strong interfaces; this is described in detail in the Experimental Section. Figure 1c–e shows the scanning electron microscopy (SEM) images of the TMHA‐based dielectric layer. Evidently, the TMHAs were uniformly distributed with a 1:1 diameter‐to‐height ratio (Figure 1c). Figure 1d shows the cross‐section SEM images of the TMHA‐based tactile sensors, and the enlarged SEM image shows a gap of 50 µm. Clearly, the graphene/PDMS TMHA did not separate from the precured layer when the dielectric layer was twisted, bent, or stretched (Figure S3, Supporting Information). As shown in Figure 1e, when the top layer was roughly peeled from one corner, the discrete rupture mode and asymmetric tilted structure of the TMHA maintained local stability to prevent continuous, catastrophic, and brittle failure. Hence, the biomimetic TMHA structure with the all‐graphene‐PDMS material system can be used as the dielectric layer of a tactile sensor to sustain and sense torque.

### 2.2 COMSOL Simulations of the TMHA‐Based Tactile Sensors and Sensing Mechanism

**Figure 2:**
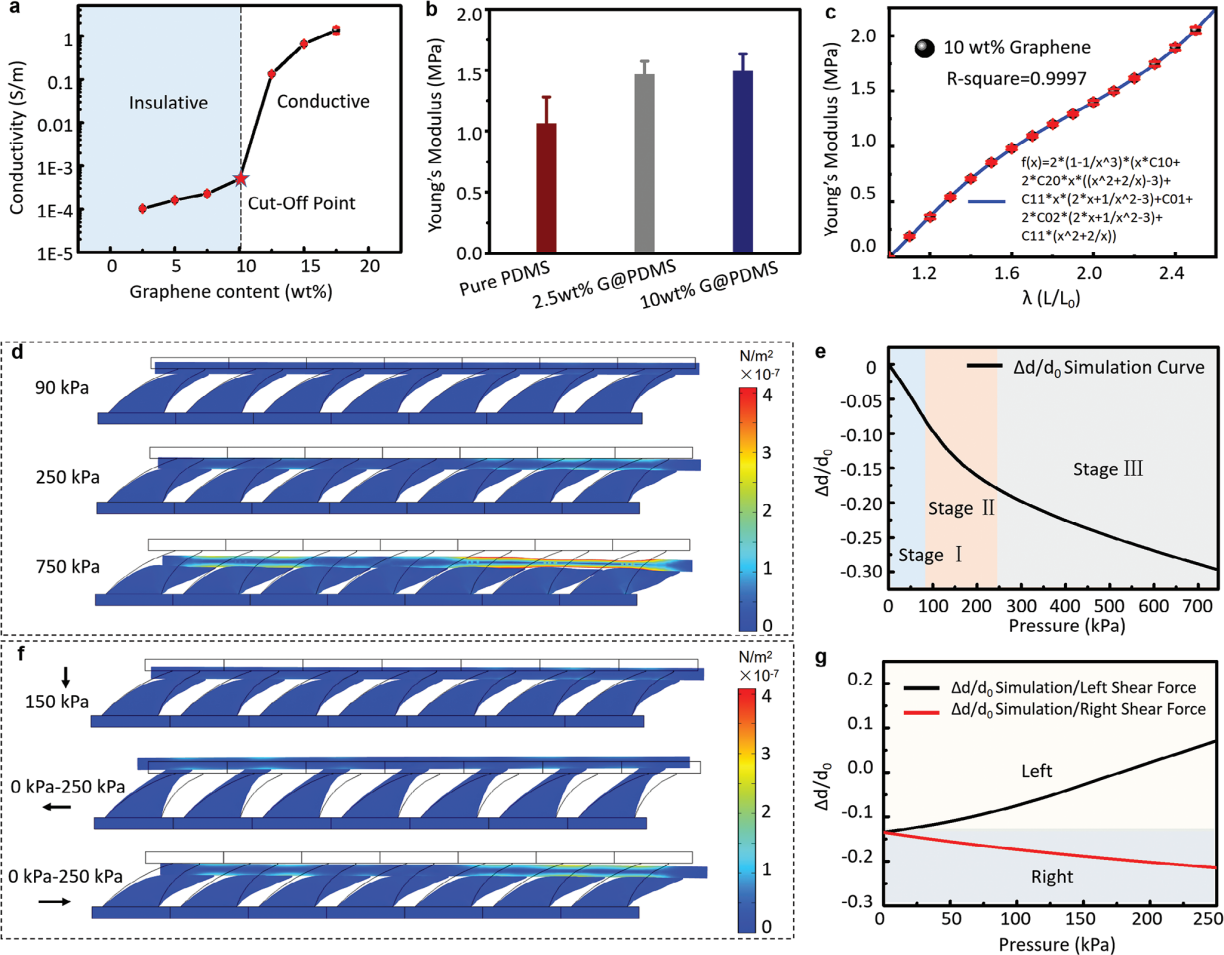
COMSOL simulations of the TMHA‐based tactile sensors. a) Conductivity curve of graphene–PDMS films, which varies with the concentration of graphene. b) Young's moduli of pure PDMS, 2.5 wt% graphene/PDMS, and 10 wt% graphene/PDMS. c) Mooney–Rivlin fitting curve of 10 wt% graphene/PDMS. d) COMSOL simulations of deformation and stress distribution of the TMHA‐based tactile sensors under a pressure of 0–750 kPa. e) Relationship between electrode displacement and pressure between 0 and 750 kPa. f) COMSOL simulations of deformation and stress distribution of the TMHA‐based tactile sensors with directional shear force from 0 to 250 kPa under a pressure of 150 kPa. g) Relationship between electrode displacement and directional shear force pressure from 0 to 250 kPa under a pressure of 150 kPa.

The deformation of the TMHAs was simulated to investigate the response of the dielectric layer gap when subjected to pressure and bidirectional shear force loading on the tactile sensor. The resistance of graphene/PDMS increased with the doping concentration of graphene, and 10 wt% was considered as the cutoff point between the conducting and insulating graphene/PDMS (**Figure** **2**a). Figure 2b shows the Young's moduli of graphene/PDMS with different mass fractions. Considering its conductivity and material strength, 10 wt% graphene/PDMS was chosen as the dielectric layer for the TMHA‐based tactile sensors in this study. The graphene/PDMS composite is a hyperelastic material whose strain changes nonlinearly with stress. The length variation ratio (λ) is defined as:

(1) $$\lambda \;=\frac{L}{L_{0}}\;$$

where *L* is the length after deformation, and *L*_0_ is the initial length. A Mooney‐Rivlin model with five parameters was used to fit the Young's modulus curve. The relationship of the pressure (*P*) with λ is as follows:

(2) $$\begin{array}{ccc} P & = & 2\left(1-\lambda^{-3}\right)(C_{10}\lambda +2C_{20}\lambda \left(\lambda^{2}+2\lambda^{-1}-3\right)\\ & & -\;C_{11}\lambda \left(2\lambda +\lambda^{-2}-3\right)+C_{01}+2C_{02}\left(2\lambda +\lambda^{-2}-3\right)\\ & & +\;C_{11}\left(\lambda^{2}+2\lambda^{-1}\right))\left(\mathrm{MPa}\right) \end{array}$$

where *C*_10_ = 0.8857, *C*_20_ = 0.1585, *C*_11_ = ‐0.5372, *C*_01_ = 1.002, and *C*_02_ = 0.3147. As shown in Figure 2c, the Mooney‐Rivlin fitting curve agreed with the experimental data (*R* ^2^ > 0.9997). The fitting curve was then used for the deformation simulation of the TMHA when it was loaded by pressure and shear force.

The models simulated by the COMSOL software are shown in Figure 2d–g; evidently, as the pressure increased, the distance decreased continuously. Because of the TMHA structure, three linear intervals were observed when the applied pressure ranged from 0 to 700 kPa. Note that if the applied pressure is low, only the top taper of the TMHA deforms (Figure 2e, Stage I). Subsequently, with increasing pressure, the entire TMHA undergoes deformations (Figure 2e, Stage II). Finally, the entire TMHA is compressed (Figure 2e, Stage III). As shown in Figure 2f, TMHA produced different deformations under a pressure of 150 kPa and bidirectional shear. When the shear direction was the same as that of the TMHA structure, the distance between the dielectric layers decreased. Interestingly, when the shear direction was opposite to that of the TMHA structure, the distance between the dielectric layers increased (Figure 2g). Note that the relative change of TMHA height (Δ*d*/*d*_0_) exhibits an opposite trend when the shear force is loaded in different directions. When the same shear force was applied to the left and right, the Δ*d*/*d*_0_ of the hemispheres and pyramids exhibited a linear and simultaneous decrease (Figure S4, Supporting Information) because of the symmetrical structure. Therefore, the response tendency of the TMHA‐based dielectric layer gap, which differs from that of conventional structures, can be used to distinguish the direction of the shear force after deformation simulation. Furthermore, the ratio of the diameter to the height and angle of inclination must be investigated for improving THMAs.

### 2.3 Pressure Sensing Properties of the TMHA‐Based Capacitive Sensor

**Figure 3:**
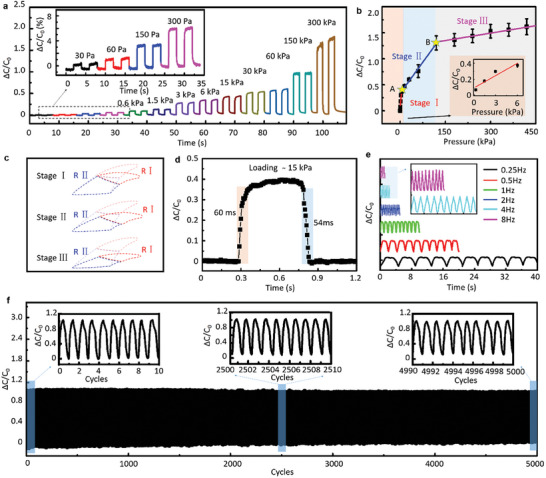
Pressure sensing properties of the TMHA‐based tactile sensor. a) Relationship between relative capacity changes and applied pressure from 30 Pa to 300 kPa. b) Sensitivity of the pressure sensing performance. c) Working mechanism of pressure sensing. d) Response and recovery time of the sensors. e) Relative capacity changes of the sensors under different frequencies. f) Stability of the sensors.

The TMHA‐based capacitive sensor demonstrated a wide pressure‐response range and high sensitivity in the low‐pressure regime. **Figure** **3**a shows the relationship between the relative capacitive change (Δ*C*/*C*_0_) and applied pressure from 30 Pa to 300 kPa. The magnified view in Figure 3a indicates that the detection limit of the sensor was 30 Pa. Δ*C*/*C*_0_ increased with increasing pressure in three linear intervals (Figure 3b). The pressure sensing mechanism of the tactile sensors is shown in Figure 3c. Note that the capacity of a capacitive pressure sensor is related to the distance between the dielectric layers. Increasing the pressure decreased the gap, which enhanced Δ*C*/*C*_0_. In addition, the TMHA unit has an asymmetric structure similar to that of a tilted circular cone. The units can be divided into RI and RII units (Figure S5, Supporting Information). When the pressure ˂6 kPa, the tapered RI produced deformations with a sensitivity of 0.0513 kPa^‐1^, corresponding to Stage I in Figure 3c. When the pressure reached 120 kPa, the entire unit (RI and RII) produced deformations with a sensitivity of 0.0079 kPa^‐1^, corresponding to Stage II in Figure 3c. When the pressure exceeded 120 kPa, the entire unit (RI and RII) was compressed, and the sensitivity was 0.0009 kPa^‐1^, corresponding to Stage III in Figure 3c. The linear fitting formulas are as follows:

(3) $$R\;=\left\{\begin{array}{c} 0.092\;+\;0.0513\times P,\;P\;<6\;kPa\;\left(R_{1}^{2}=0.862\right)\\ \;0.336\;+\;0.0079\times P,6\;\leq \;P<120\;kPa\;\left(R_{2}^{2}=0.990\right)\\ 1.213\;+\;0.0009\times P,\;P\geq 120\;kPa\;\left(R_{3}^{2}=0.981\right) \end{array}\right.\;$$

where *R* is the sensor response. Moreover, the experimental and simulation results showed good agreement (Figure S6, Supporting Information). The wide range of pressure responses exhibited by the capacitive sensor was attributed to the sustained performance of the TMHA‐based dielectric layer (Table S1, Supporting Information). In contrast, the high sensitivity resulted from the significant deformation of RI in low‐pressure conditions. Notably, a curve with two cutoff points (A and B) was observed (Figure 3b), which may have been proportionately linked to the RI. Therefore, future investigations into the TMHA morphology are necessary to estimate the cutoff point of the response curve.

As shown in Figure 3d, the response and recovery times of the pressure sensor at 15 kPa were 60 and 54 ms, respectively. Owing to the fast response and recovery times, the device can quickly detect pressure changes, and the pressure‐sensing performance is not affected by the frequency, as shown in Figure 3e. The stability of the sensor against different conditions is shown in Figures S7–S10 (Supporting Information). Due to the packaging process, humidity has little influence on the performance of the sensor (Figure S7, Supporting Information). With the temperature increasing, there is little influence on the pressure sensing (Figure S8, Supporting Information). The relationships between temperature/mechanical deformations and capacity are shown in Figures S9 and S10 (Supporting Information). Furthermore, inducing the temperature and deformations corrections, the tactile sensor can identify the pressure and shear force more accurately. Moreover, the device exhibited long‐term stability (Figure 3f). After over 5000 cycles under the pressure range of 0–100 kPa at a frequency of 4 Hz, the device still maintained 97.768% of the initial ΔC/C_0_, and the relative change of the mean value was ‐1.269% (Figure S11, Supporting Information).

### 2.4 Sensing Properties of the TMHA‐Based Capacitive Sensor to Shear Force

**Figure 4:**
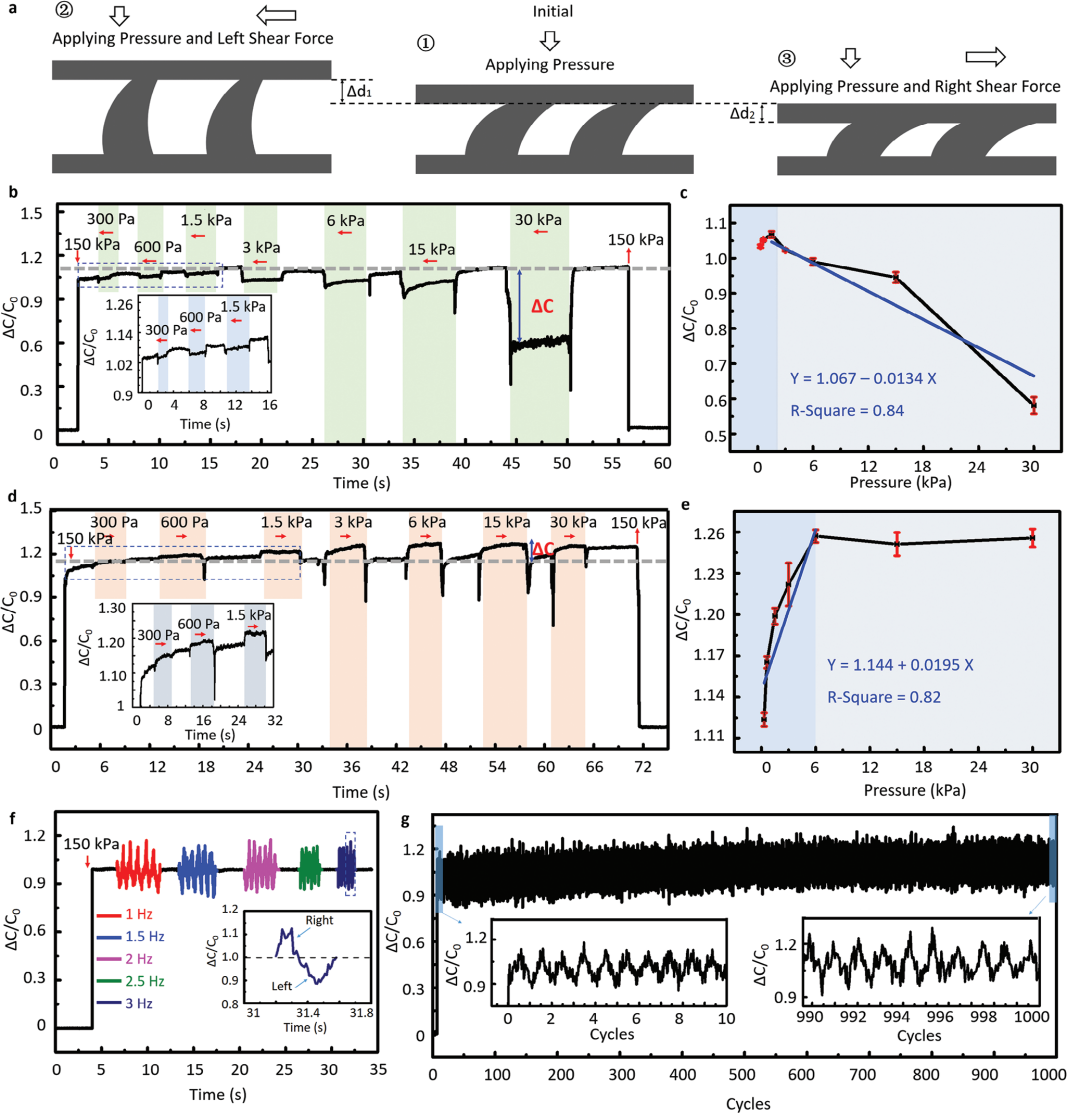
Shear force sensing properties of the TMHA‐based tactile sensor. a) Working mechanism of pressure shear force sensing. Schematic of the capacitive sensor before and after applying pressure. b) Response of left shear force ranging from 30 Pa to 30 kPa under a pressure of 150 kPa. c) Sensitivity of left shear force. d) Response of right shear force ranging from 30 Pa to 30 kPa under a pressure of 150 kPa. e) Sensitivity of right shear force. f) Frequency response of dynamic shear force. g) Durability of the sensor over 1000 cycles.

Simulation of the dielectric layer gap under a directional shear force (Figure 2g) indicated that the signal tendency of the TMHA‐based capacitive sensor could distinguish the shear force. The shear sensing mechanism of the tactile sensors is illustrated in **Figure** **4**a, and the schematic diagram of the force loading is illustrated in Figure S12 (Supporting Information). Initially, a 150 kPa load was placed on the TMHA‐based tactile sensor. Note that owing to the asymmetric structure of TMHA, the induced bending moment of TMHA is anisotropic when the tactile sensors respond to different shear directions. If the shear force is applied to the left (opposite to the TMHA structure), the induced bending moment is weaker than the bending stiffness, resulting in an increasing gap in the dielectric layer.^[^ ^43^, ^44^ ^]^ Conversely, when the shear force is applied to the right (similar to the TMHA structure), the induced bending moment is larger than the bending stiffness, resulting in a decreased gap in the dielectric layer. Hence, under a load of 150 kPa, the left shear force decreased the capacity of the tactile sensor, whereas the right shear force increased it. Figure 4b shows the left sensing properties of the tactile sensors. The capacitance changes were relative to the magnitudes of the left shear forces. As shown in Figure 4c, a linear relationship is observed between the left shear force magnitudes and relative capacitance changes, and the sensitivity is ‐0.0134 kPa^‐1^. The TMHA approaches tensile saturation when the load exceeds 30 kPa. Figure 4d shows the right sensing properties of the tactile sensors. A linear relationship is observed between the right shear force magnitudes and relative capacitance changes in the load range of 0–6 kPa, and the sensitivity is 0.0195 kPa^‐1^ (Figure 4e). When the load (right) exceeds 6 kPa, the deformation of TMHA approaches compression saturation. Therefore, the maximum detection limits of the left and right shear forces are 30 and 6 kPa, respectively (**Table** **1**).

**Table 1:** The relationships between Δ*C*/*C*_0_ of the tactile sensor and shear forces.

Direction	Range	Linear fit	*R* ^2^
Left	1.5–30 kPa	*Y* = 1.067‐0.0134 *X*	0.84
Right	300 Pa–6 kPa	*Y* = 1.144+0.0195 *X*	0.82

Because of the fast response and recovery times, the dynamic shear force was measured, as shown in Figure 4f. The brush provided a constant force of 150 kPa and reciprocated on the device. TMHA‐based tactile sensors could quickly detect the direction of the shear force, and their sensing properties were insensitive to frequency. The durability of the sensor against dynamic shear force was evaluated over 1000 cycles at 2 Hz, as shown in Figure 4g. The mean value of the left force peak changes from 1.155 ± 0.026 to 1.222 ± 0.037 after 1000 cycles, and the relative change of the mean value is ‐5.801%. The mean value of the left force peak changes from 0.889 ± 0.037 to 0.971 ± 0.021 after 1000 cycles, and the relative change of the mean value is ‐9.224% (Figure S13, Supporting Information). Therefore, the sensor has good stability for detecting shear force. These results indicate that the sensor has a stable response to dynamic shear forces over a long period of time. Hence, sensors based on the TMHA have the potential to be applied in the field of human–machine interactions as tactile interfaces.

### 2.5 Applications of the TMHA‐Based Tactile Sensors in Human–Dexterous–Hand Interactions

**Figure 5:**
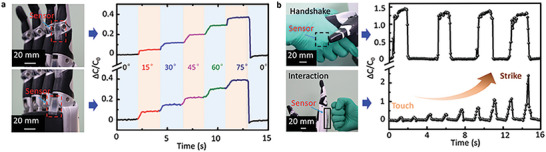
Applications of the TMHA‐based tactile sensors integrated with a dexterous hand. a) TMHA‐based tactile sensors integrated with the joints of a dexterous hand for bending sensing. b) TMHA‐based tactile sensors integrated with the palm and back of a dexterous hand for enhancing interactions.

The TMHA‐based tactile sensor exhibits the potential for integration into a dexterous hand, thus enabling human–machine interaction through its flexibility, sensitivity, and conformability. Some applications of dexterous hands integrated with TMHA‐based tactile sensors are shown in **Figure** **5**. As shown in Figure 5a, the TMHA‐based tactile sensors were attached to the distal interphalangeal and metacarpophalangeal joints, which enhance intelligence during human–machine interactions. The dexterous hands were controlled through feedback from the TMHA‐based tactile sensors via angle sensing.

When the bending angle of the distal interphalangeal joint increased from 0° to 15°, 30°, 45°, 60°, and 75°, Δ*C*/*C*_0_ increased from 0.008 to 0.086, 0.151, 0.225, 0.312, and 0.387, respectively. Similarly, when the bending angle of the metacarpophalangeal joint increased from 0° to 15°, 30°, 45°, 60°, and 75°, Δ*C*/*C*_0_ increased from 0.003 to 0.053, 0.121, 0.204, 0.295, and 0.348, respectively, as shown in **Table** **2**. The curves indicate that the sensors respond quickly to changes in the angles of the dexterous fingers. The TMHA‐based sensors were integrated into the palm and back of a dexterous hand to enhance safety during human–machine interactions, as shown in Figure 5b. The change in Δ*C*/*C*_0_ during the handshake process indicates the pressure between the dexterous and human hands. The position and force in the handshake process could be adjusted to make it more comfortable through feedback on the angle of the joint and the pressure of the palm. The change in Δ*C*/*C*_0_ during a dexterous hand attack by different forces shows the process from touch to strike, indicating the friendliness of the interactive object.

**Table 2:** Variation of Δ*C*/*C*_0_ of the sensor integrated in different joints with the bending angle.

Bending angles	0°	15°	30°	45°	60°	75°
Interphalangeal joint	0.008	0.086	0.151	0.225	0.312	0.387
Metacarpophalangeal joint	0.003	0.053	0.121	0.204	0.295	0.348

### 2.6 Application of TMHA‐Based Tactile Sensors in Robot Grasping

**Figure 6:**
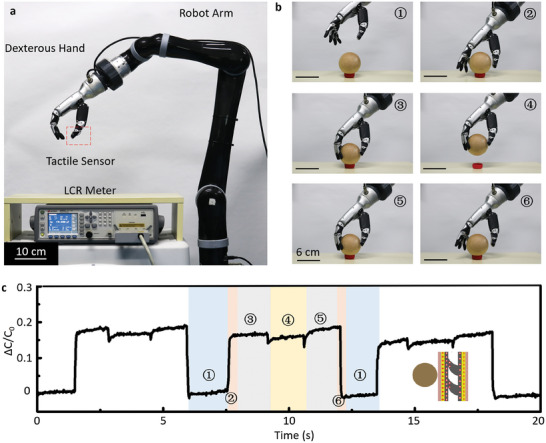
Applications of the TMHA‐based tactile sensors integrated with robot hand for grasping experiments. a) Photograph of the experimental system. b) Stepwise process of robot hand during grasping wooden ball: approaching, touching, grasping, lifting, lowering, and releasing. c) Sensing performance of the TMHA‐based tactile sensors during several grasping experiments.

To enhance the robot perception and precision in fine manipulation tasks, a TMHA‐based tactile sensor was mounted on the thumb of the robot hand as tactile e‐skin. The experiments replicated the process of robot grasping, as shown in **Figure** **6**a, and an LCR meter was used to record the capacitance of the device during the grasping process. The robotic hand grasped a wooden ball weighing 114 g, driven by a robotic arm. The entire grasping process can be explained in a stepwise manner, as shown in Figure 6b, including approaching, touching, grasping, lifting, loosening, and releasing.

The stability of the grasp is enhanced by integrating a robotic thumb with a flexible tactile sensor, which transforms point contact into surface contact between a rigid robotic finger and the ball. First, the robotic hand approaches a wooden ball. Next, the robot hand touches the wooden ball, and Δ*C*/*C*_0_ immediately increases in response to the applied pressure. When the robotic hand firmly grasps the wooden ball, Δ*C*/*C*_0_ remains constant. Next, the wooden ball is lifted, and the weight of the ball provides a shear force. The direction of the shear force is opposite to that of TMHA, thus contributing to a decrease in Δ*C*/*C*_0_. Subsequently, the wooden ball is returned to the table, and the shear force disappears, thereby resulting in an increase in Δ*C*/*C*_0_. Finally, the wooden ball is released by the robot hand, and the TMHA‐based tactile sensor recovers to its initial state. The one‐cycle curve in Figure 6c shows a one‐to‐one correspondence of Figure 6b and some cycles demonstrate the repeatability of the TMHA‐based tactile sensors for sensing pressure and shear force. When the dexterous hand performs the grasping task, especially when the gravity direction of the object is inconsistent with the pressure direction of the manipulator, the shear force can guarantee the stability of the grasping. Moreover, in the process of dynamic grasping, the real‐time monitoring of the shear force can also help to predict whether the object has a sliding trend. In future, inducing some parameters containing the magnitude and direction of shear force can help the dexterous hand predict the movement trend of the object in time during high‐speed working processes.

## 3 Conclusion

In summary, TMHA‐based tactile sensors were proposed for sensing pressure and shear force. The asymmetric structure of TMHA produces different bending moments in response to different directional shear forces. The TMHA‐based tactile sensors exhibited high sensitivity and stability in pressure and shear force sensing. In addition, the robot and dexterous hand integrated with TMHA‐based tactile sensors enhanced the interaction performance and safety. Grasping experiments demonstrated the potential for application in robot tactile feedback systems. This type of tactile sensor provides a new direction for designing shear force sensors and can be used to develop human–machine interfaces.

## 4 Experimental Section

### PVA–Water–Ethanol Solution

PVA particles (J0405‐500G, Hefei BASF Biotechnology Co., Ltd., China) were mixed with deionized water in a weight ratio of 1:9. The mixture was stirred at 500 rpm at 40 °C for 4 h using a magnetron mixer (MS‐H‐Pro^A^, DLAB Scientific Inc., USA) to prepare a homogeneous PVA–water solution. A 10% PVA–water solution was mixed with ethanol in a weight ratio of 3:2. The mixture was stirred at 500 rpm for 2 h at 25 °C using a magnetron mixer to prepare a homogeneous PVA–water–ethanol solution (Figure S14, Supporting Information).

### Graphene–PDMS Solution

PDMS (Sylgard 184, Dow Corning Corporation, USA) with a weight ratio of 10:1 was weighed using a weighing balance (GL224I‐1SCN, Sartorius, Germany). Graphene (HGP‐50, Qingdao Yanhai Carbon Material Co., Ltd, China), PDMS, and n‐hexane (Liaoning Xinxing Reagent Co., Ltd, China) were mixed at a ratio of 1 g:9 g:200 mL in a beaker and stirred for 2 min using a glass rod. The mixture was sonicated for 1 h in a water bath at 69 °C using an ultrasound machine (YM‐020S, Shenzhen Fangao Microelectronics Co., Ltd, China). The mixture was stirred in cold water for 2 min to prepare a homogeneous graphene–PDMS solution (Figure S15, Supporting Information). N‐hexane was used to disperse graphene to obtain a uniform mixture (Figure S16a, Supporting Information). The graphene/PDMS obtained by direct mixing (Figure S16b, Supporting Information) had an uneven graphene distribution.

### Microhair Array Fabrication

1) The TMHAs were fabricated using a TPP system (Photonic Professional GT, Nanoscribe GT2, Germany) with a 25× oil immersion objective. After polymerization, the sample was developed with a propylene ether–methanol acetate solution for 20 min and cleaned with isopropanol for 3 min. 2) TMHA was used as the mold, and PDMS at a weight ratio of 10:1 was poured onto the molds and heated at 80 °C for 2 h on a heating plate (PC‐600D; Corning, USA). The cured PDMS was peeled off the molds to obtain PDMS films with micropits. 3) The PDMS films with micropits were used as molds, and an ultraviolet (UV)‐curable adhesive (NOA 74, Norland Products, Inc., USA) was poured onto the molds and vacuumed in a vacuum oven (DZF‐6012, Shanghai Yiheng Technical Co., Ltd., China) for 10 min. The samples were processed at 15 W for 10 min in a UV curing box (GHS‐LFDA3501100, Shenzhen Guanghuashi Technology Co., Ltd., China). The cured UV‐curable adhesive was peeled off the molds to obtain UV‐curable adhesive films with TMHA. 4) UV‐curable adhesive films were used as molds, and the PVA–water–ethanol solution was poured onto the molds and vacuumized in a vacuum oven for 2 min. The sample was then heated to 100 °C for 20 min on a heating plate. PVA was peeled off the molds to obtain PVA films with micropits (Figure S17, Supporting Information). 5) The PVA films were used as molds, and the graphene/PDMS solution was poured onto the molds and vacuumed in a vacuum oven for 20 min. The sample was then heated to 100 °C for 1 h on a heating plate. Graphene/PDMS was peeled off the molds to obtain graphene/PDMS films with TMHA.

### Sensor Package

Dielectric layer encapsulation: 1) A 250 µm graphene/PDMS solution was scraped onto a glass substrate. 2) The graphene/PDMS films were cured at 100 °C for 2.5 min on a heating plate. 3) The graphene/PDMS films with TMHA were inverted on the surface of the fabricated graphene/PDMS film to ensure full contact between the hair‐like array and the precured film. 4) The sample was heated at 100 °C for 1 h on a heating plate to prepare a bonded graphene/PDMS dielectric layer. Conductive layer: A thin, highly conductive layer of Cr (5 nm) and Au (50 nm) was deposited on the cleaned PET substrate using a magnetron ion sputtering system (TRP450, Sky Technology Development Co. Ltd., China). Sensor package: Two conductive layers were connected to the dielectric layer using conductive silver glue (3813, Ausbond, China), and PDMS was cast onto the sensor as the packaging layer.

### Characterization

The conductivities of the graphene–PDMS films were evaluated using a digital multimeter (2400, Keithley, USA). The Young's moduli of pure PDMS, 2.5 wt% graphene–PDMS, and 10 wt% graphene–PDMS were tested using a tensile testing machine (1^ST^, Tinius Olsen, USA). The performance of the TMHA‐based sensors was evaluated using the tensile testing machine and an LCR meter (E4980AL, Keysight, USA). A robot arm (Jaco2, Kinova, Canada) and a dexterous hand (RH56DFX, Inspired‐robots, China) were used to verify the potential application capabilities of the sensors. COMSOL Multiphysics software was used for the simulations. Optical images were captured using a SONY FDR‐AX45 video camera. A scanning electron microscope (Quattro S, Thermo Fisher Scientific, USA) was used to observe the PVA micropits, graphene/PDMS TMHA, and fabrication process of the sensor.

## Conflict of Interest

The authors declare no conflict of interest.

## Supporting information

Supporting Information

## Data Availability

The data that support the findings of this study are available from the corresponding author upon reasonable request.
