# Supplementary material for: Skin‐Inspired Capacitive Flexible Tactile Sensor with an Asymmetric Structure for Detecting Directional Shear Forces
Source: Adv Sci (Weinh). 2023 Dec 7;11(6):2305883. doi: 10.1002/advs.202305883 (PMC10853706; doi:10.1002/advs.202305883)
Supplement: Supplementary file 1 — Supporting Information [file ADVS-11-2305883-s001.pdf]

ADVANCED SCIENCE
Open Access

Supporting Information

for *Adv. Sci.*, DOI 10.1002/advs.202305883

Skin-Inspired Capacitive Flexible Tactile Sensor with an Asymmetric Structure for Detecting Directional Shear Forces

Haibo Yu, Hongji Guo, Jingang Wang, Tianming Zhao, Wuhao Zou, Peilin Zhou, Zhuang Xu, Yuzhao Zhang, Jianchen Zheng, Ya Zhong, Xiaoduo Wang and Lianqing Liu**

Supporting Information

Skin-Inspired Capacitive Flexible Tactile Sensor with an Asymmetric Structure for Detecting Directional Shear Forces

Haibo Yu Hongji Guo, Jingang Wang, Tianming Zhao, Wuhao Zou, Peilin Zhou, Zhuang Xu, Yuzhao Zhang, Jianchen Zheng, Ya Zhong, Xiaoduo Wang and Lianqing Liu**

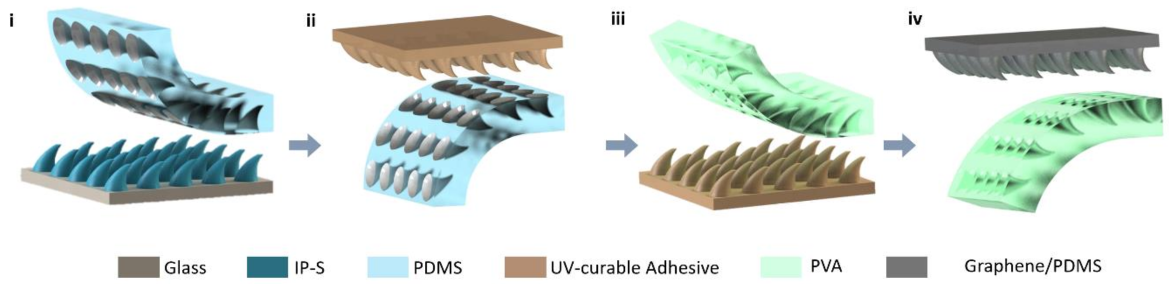

Figure S1. Processes of the four replica templates. First, the PDMS was used to turn the IP-S TMHA mold (i). Second, the ultraviolet (UV)-curable adhesive was made into the TMHA when the PDMS was used as a mold (ii). The bond strength between IP-S and glass is weak, and the hardness of IP-S is much less than the UV-curable adhesive. Thanks to the above process, the UV-curable adhesive TMHA could be turned multiple times instead of the IP-S TMHA, with only once TPP process. Third, the PVA-water-ethanol solution was used to turn the UV-curable adhesive TMHAs mold (iii). Forth, the mixture of graphene and PDMS was made into the TMHA when the PVA was used as a mold (iv).

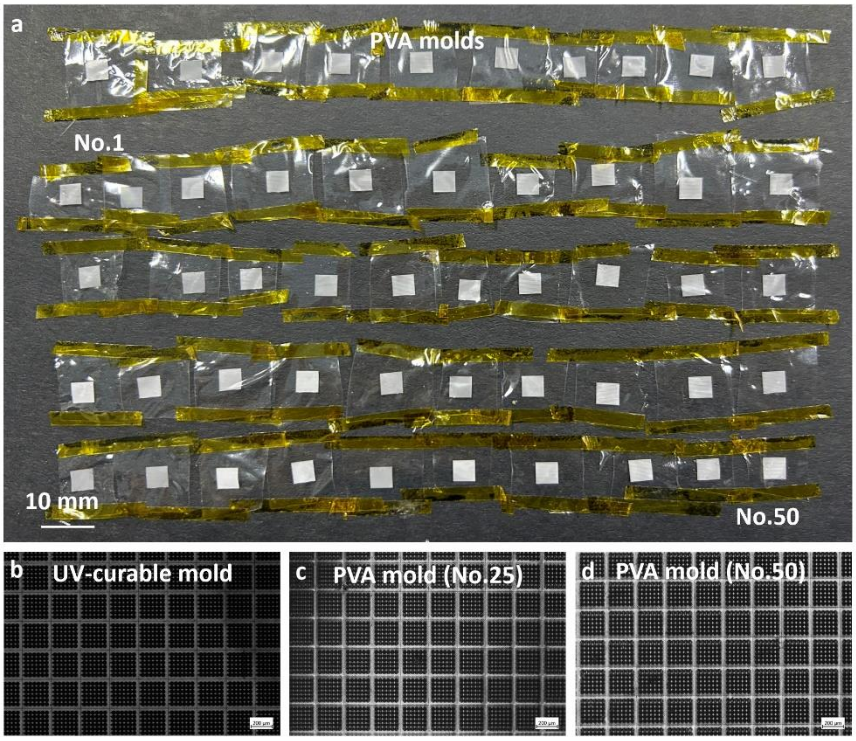

Figure S2. UV-curable adhesive mold and PVA molds. the UV-curable adhesive TMHA remains intact after 50 mold turns, thus can be turned multiple times instead of the IP-S TMHA. PVA mold also remains intact after 25 mold turns and 50 mold turns.

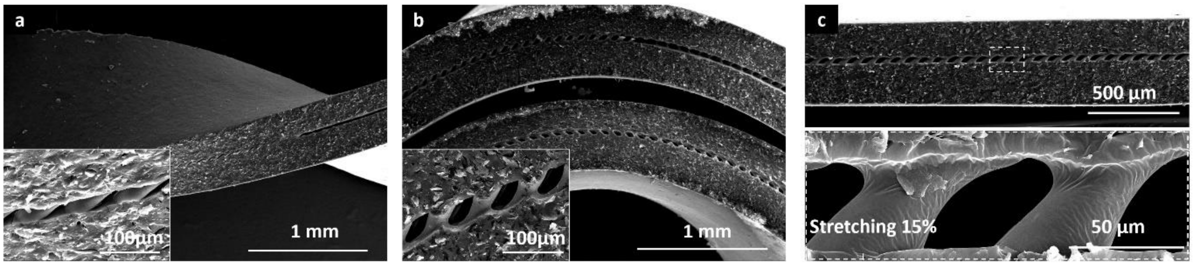

Figure S3. (a) SEM of the packaged sensor dielectric layer in twisting. (b) SEM of the packaged sensor dielectric layer in bending. (c) SEM of the packaged sensor dielectric layer in stretching 15%.

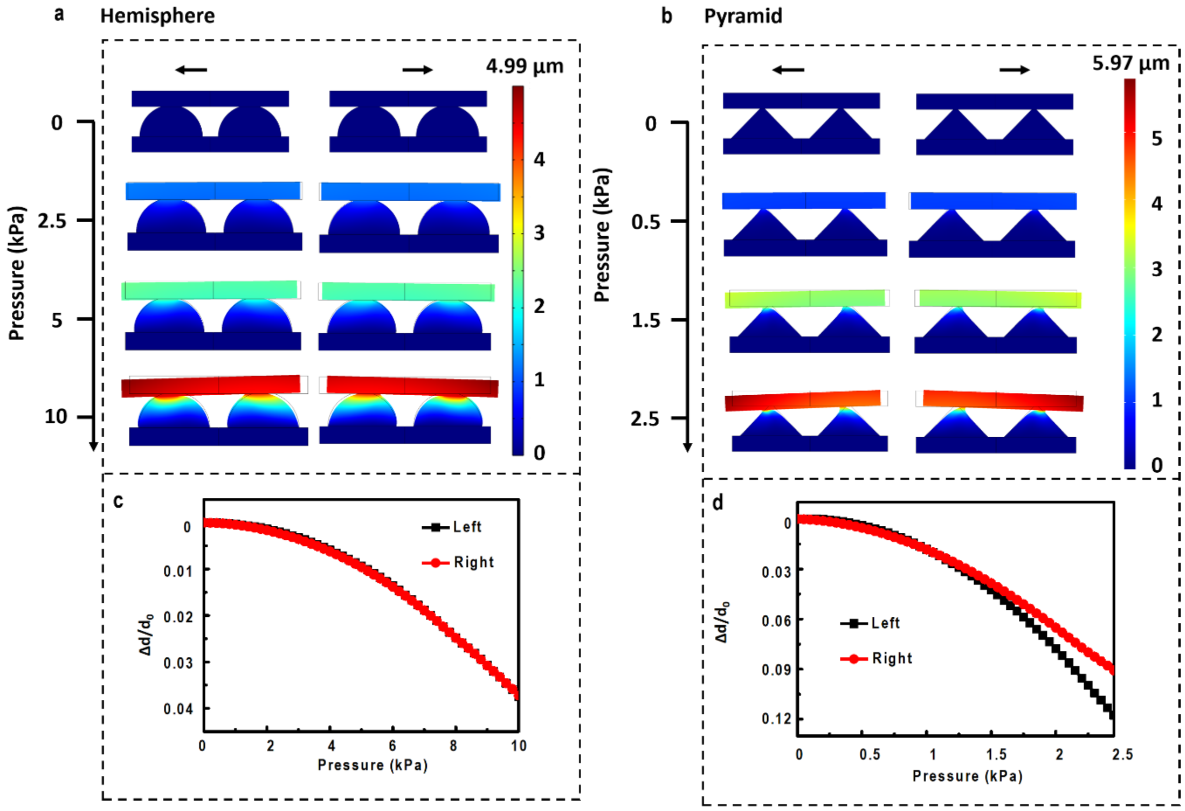

Figure S4. Simulation of the deformation of hemispheres and pyramids under shear forces in different directions.

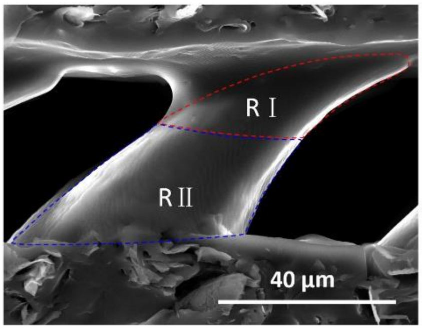

Figure S5. SEM image of a tilted microhair in the dielectric layer, where R I is the bonding region with the upper layer, and R II is the free region of the root.

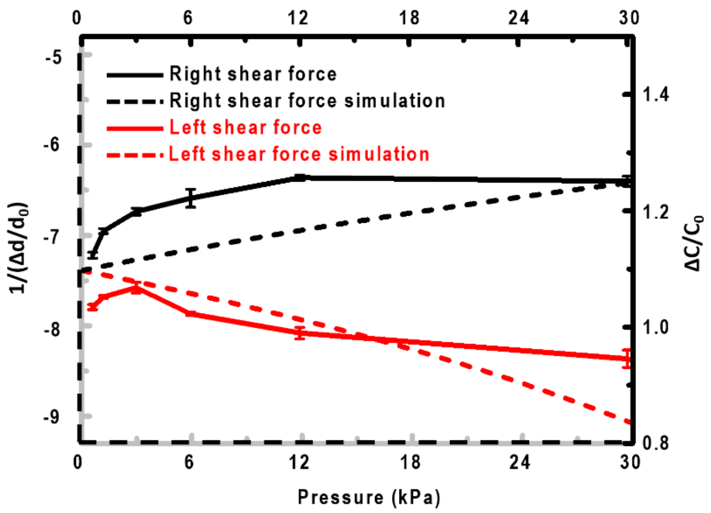

Figure S6. The simulation ($1/(d/d_0)$) and test (C/C_0).

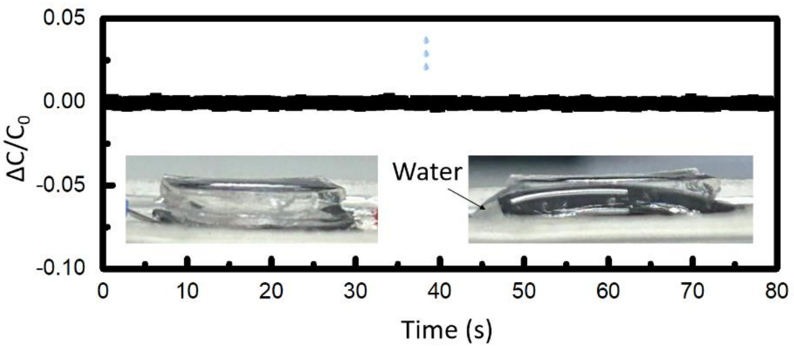

Figure S7. The $\Delta C/C_0$ of the sensor with humidity

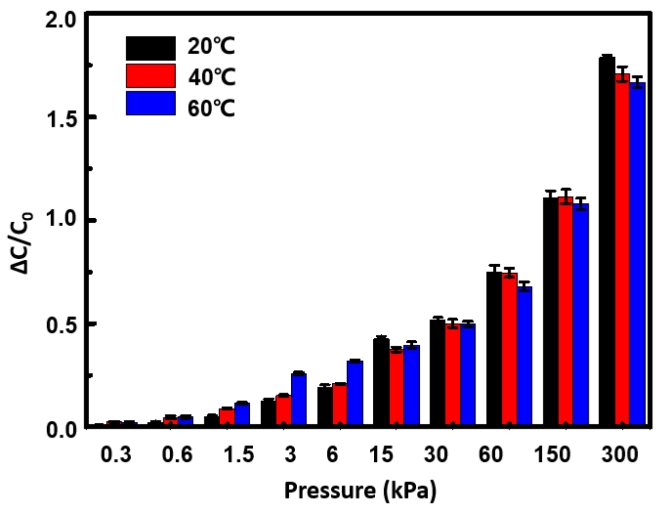

Figure S8. Pressure sensing performance against different temperature.

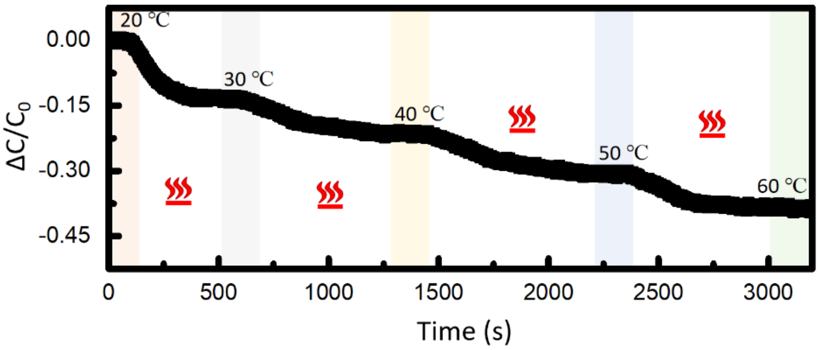

Figure S9. The $\Delta C/C_0$ of the sensor against different temperature.

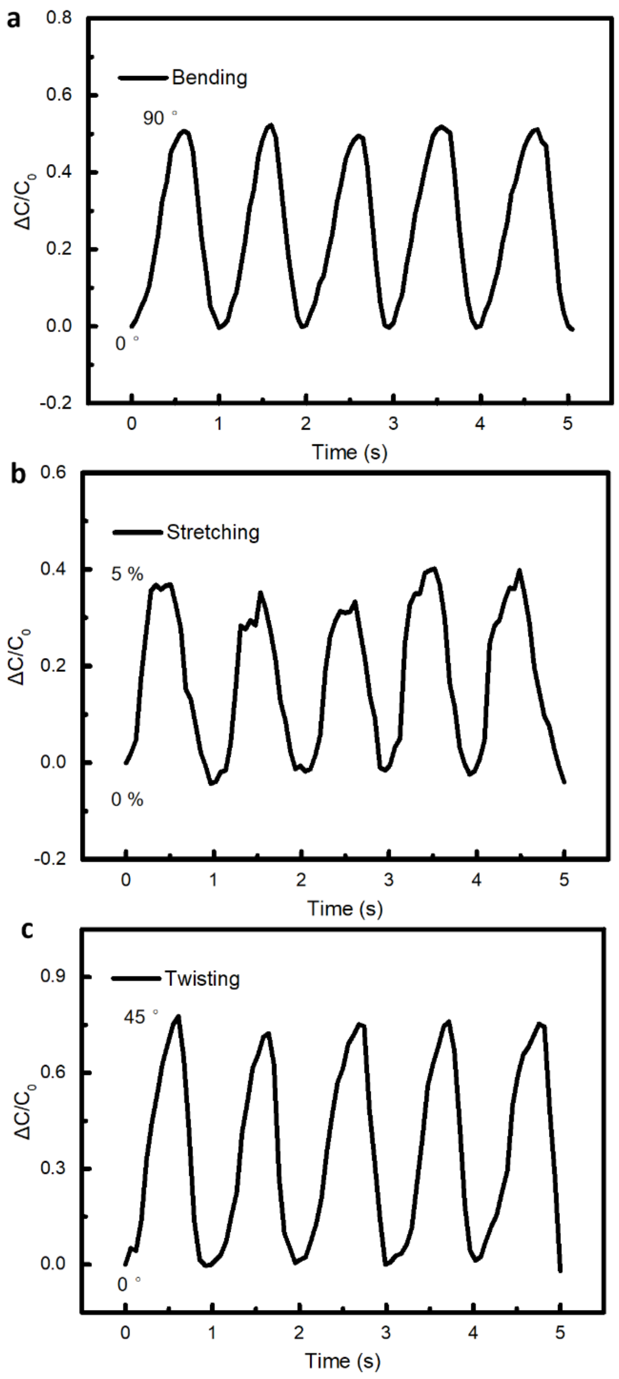

Figure S10. The $\Delta C/C_0$ of the sensor after bending (a), stretching (b), and twisting (c).

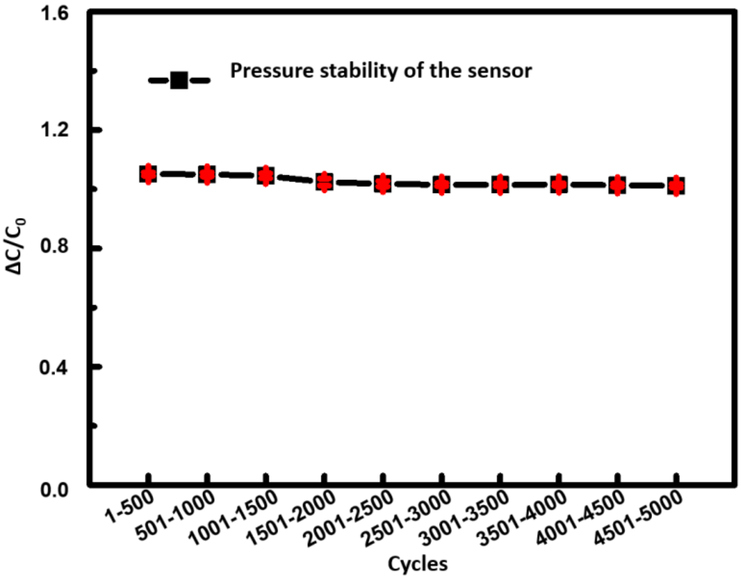

Figure S11. Pressure stability of the sensors. The mean value of the peak value changes from 1.024±0.005 to 1.011±0.006 after 5000 cycles, and the relative change of the mean value is -1.269%.

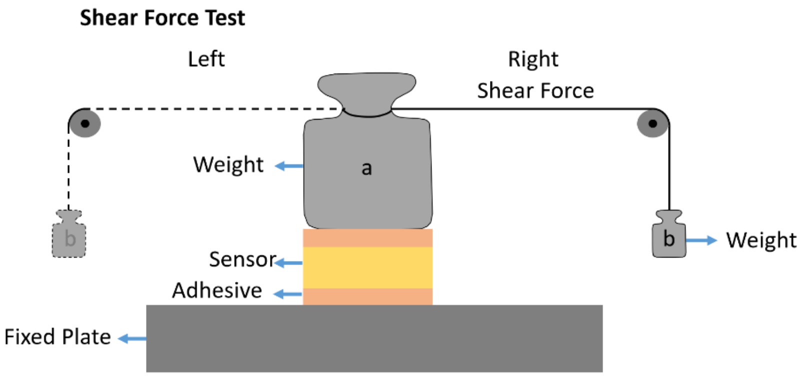

Figure S12. Schematic diagram of the force loading.

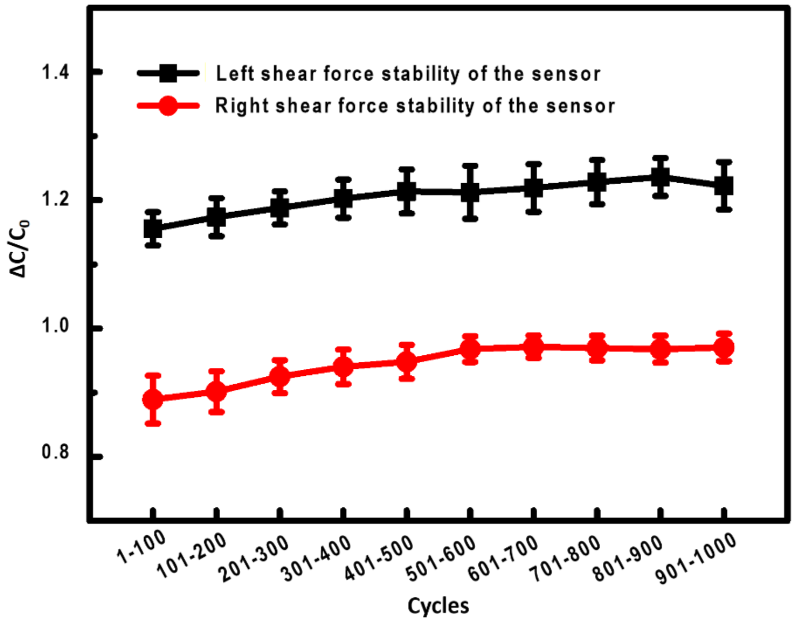

Figure S13. Shear force stability of the sensors.

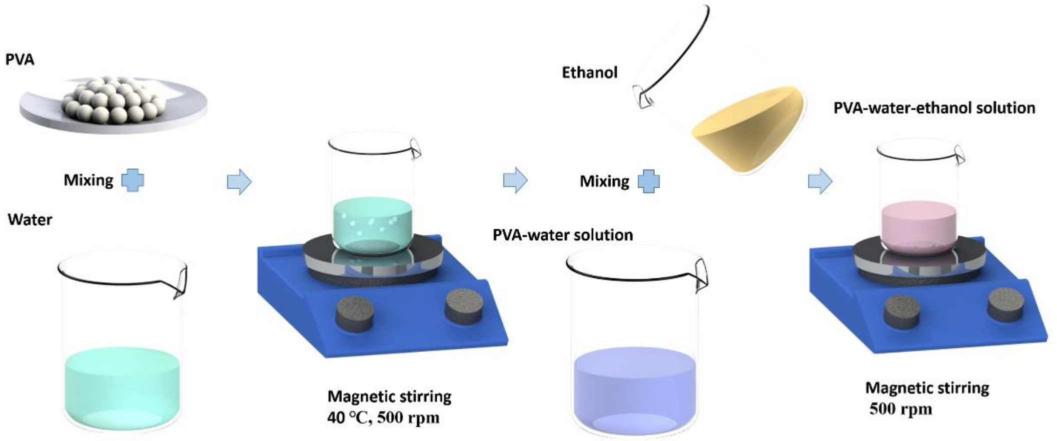

Figure S14. The PVA–water–ethanol solution was prepared by a two-step ultrasonic mixing method.

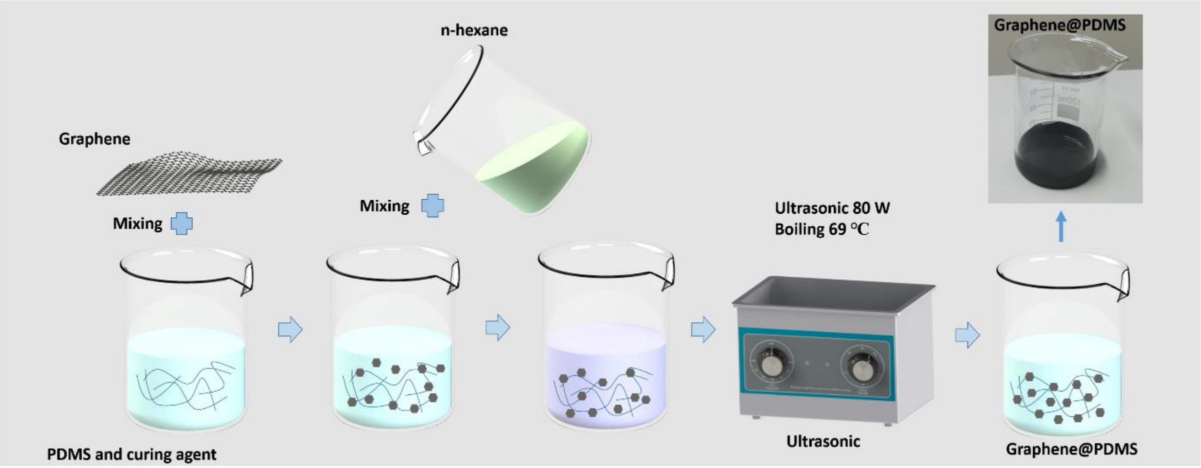

Figure S15. Preparation of graphene–PDMS hybrid logistics using n-hexane.

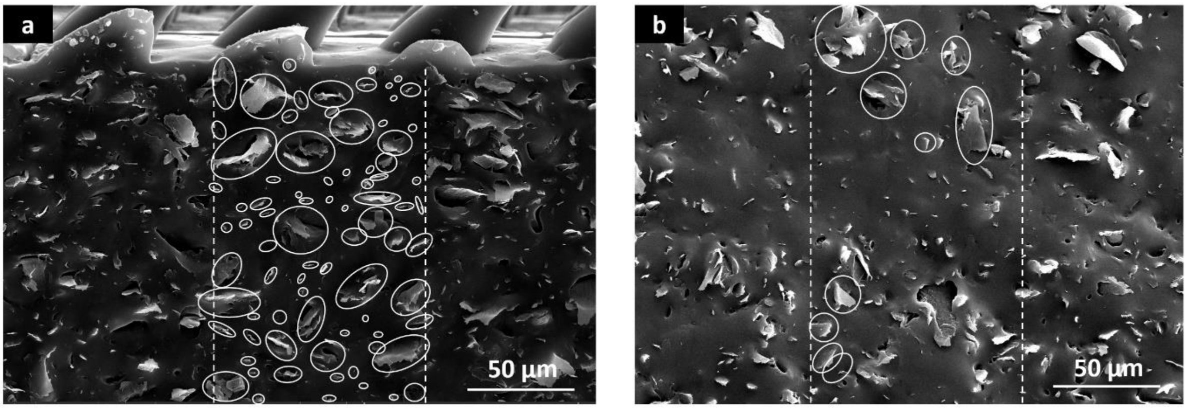

Figure S16. (a) Graphene distribution in the cross-section of graphene-PDMS films prepared using n-hexane. (b) Graphene distribution in the cross section of graphene-PDMS films prepared by direct mixing.

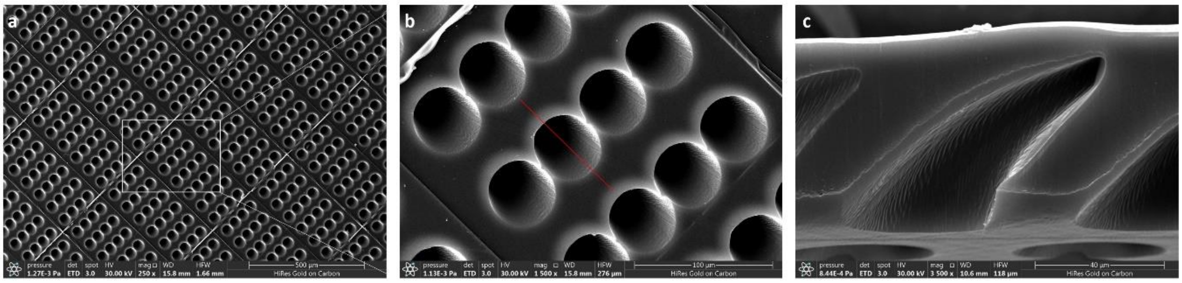

Figure S17. SEM images of the PVA micropit mold made with the microstructure of the photocuring adhesive.

Table S1. Complete performance parameters of the tactile sensor.

No.	Sensitivity	Limit	Range	Linearity	Structure	Reference
1	0.252 V N^{-1}	—	0—10 N (Z-axis)<br>0—1.6 N (X- and Y-axes)	0.956	4 Pixels	[23]
2	6.33 kPa^{-1}<br>2.31 kPa^{-1}<br>0.92 kPa^{-1}<br>1.28 N^{-1}	0.09 kPa	0.1—0.3 kPa (Normal sensitivity)<br>3.0—10.0 kPa (Normal sensitivity)<br>10.0—18.8 kPa (Normal sensitivity)<br>0—0.90 N (Shearing sensitivity)	—	3 Pixels	[24]
3	-0.2207 N^{-1}<br>-0.1976 N^{-1}	0.1 mN	0—1.1 N (Normal force)<br>-1 N—1 N (Shear force)	—	4 Optic channel	[25]
4	2.1086% N^{-1}<br>~3.2% N^{-1}	—	0—5 N (Normal force)<br>0—0.5 N (Shear force)	—	4 Pixels	[27]
5	2.65 N^{-1}<br>0.3 N^{-1}	—	0—1.2 N (Normal force)<br>0—1.0N (Shear force)	0.998<br>0.979	4 Pixels	[28]
6	0.0124 kPa^{-1}<br>0.0752 N^{-1}<br>0.0177 N^{-1}	2 Pa	0—12 kPa (Pressure)<br>0.05—0.50 N (Shear force)<br>0.05—1.30 N (Shear force)	0.95<br>0.95<br>0.96	1 Array<br>(No direction)	[32]
7	0.19±0.07 kPa^{-1}<br>0.10±0.01 kPa^{-1}<br>0.04±0.001 kPa^{-1}<br>3.0±0.5 Pa^{-1}	15 mg	0—1 kPa (Normal pressure)<br>1—10 kPa (Normal pressure)<br>10—20 kPa (Normal pressure)<br>10—20 kPa (Shear force)	—	25 Pixels	[34]
8	0.0173 kPa^{-1}<br>0.165 kPa^{-1}	—	128 Pa—44 kPa (Normal force)<br>5.28 kPa—12.9 kPa (Shear force)	—	4 Pixels	[35]
9	0.0513 kPa^{-1}<br>0.0079 kPa^{-1}<br>0.0009 kPa^{-1}<br>-0.0134 kPa^{-1}<br>0.0195 kPa^{-1}	30 Pa	0—6 kPa (Normal pressure)<br>6—120 kPa (Normal pressure)<br>＞120 kPa (Normal pressure)<br>1.5 kPa—30 kPa (Left shear force)<br>300 Pa—6 kPa (Right shear force)	0.86<br>0.99<br>0.98<br>0.84<br>0.82	1 Array<br>(Direction)	This study
